# Genetic Stability and Photosystem II Functioning of In Vitro-Recovered *Lamprocapnos spectabilis* (L.) Fukuhara After ZnO + Ag Nanoparticles or Melatonin Exposure During Vitrification—Preliminary Study

**DOI:** 10.3390/ijms262210817

**Published:** 2025-11-07

**Authors:** Dariusz Kulus, Alicja Tymoszuk, Mateusz Cichorek

**Affiliations:** 1 Laboratory of Horticulture and Landscape Architecture, Department of Biotechnology, Faculty of Agriculture and Biotechnology, Bydgoszcz University of Science and Technology, Bernardyńska 6, 85-029 Bydgoszcz, Poland; alicja.tymoszuk@pbs.edu.pl; 2 Department of Plant Physiology, Genetics and Biotechnology, Faculty of Biology and Biotechnology, University of Warmia and Mazury in Olsztyn, Oczapowskiego 1a, 10-719 Olsztyn, Poland; mateusz.cichorek@uwm.edu.pl

**Keywords:** bleeding heart, cryopreservation, encapsulation-vitrification, explant recovery, nanomaterials, photosynthetic efficiency, PVS, SCoT, synthetic seeds

## Abstract

The success of plant tissue cryopreservation strongly depends on maximizing explant survival during storage in liquid nitrogen and recovery, which requires species-specific protocol optimization and ongoing refinement. This study examined the effect of Plant Vitrification Solution 3 (PVS3) supplemented with nanoparticles (NPs) or melatonin (MEL) on the recovery of *Lamprocapnos spectabilis* (L.) Fukuhara explants after cryostorage. Treatments with ZnO + Ag NPs, as well as different MEL concentrations, were applied to evaluate their influence on explant survival, photosynthetic efficiency, and genetic stability. The highest recovery (40–44%) was obtained with PVS3 containing 50 mg L^−1^ ZnO + 0.1% Ag NPs and PVS3 supplemented with 8 mg L^−1^ MEL, which was 17.5–20% higher than in the control. These treatments, however, did not ensure the highest photosynthetic efficiency of recovered plants. PVS additives likely support recovery by slowing metabolism and reducing oxidative stress, with lower photosynthetic activity suggesting a lag phase in plastid regeneration. Using the Start Codon Targeted (SCoT) marker system, no significant genetic alterations were detected in recovered plants of any tested variant. These findings demonstrate the feasibility of optimizing cryopreservation protocols for *L. spectabilis* and encourage further research on combined NPs and MEL treatments or alternative nanocarriers.

## 1. Introduction

Bleeding heart (*Lamprocapnos spectabilis* [L.] Fukuhara) is a long-lived, dormant, perennial of the Fumarieae Dumort. tribe (Papaveraceae), native to East Asia, particularly China and Japan [1]. It is widely recognized for its striking heart-shaped ornamental flowers with different colors (pink for wild form; shades ranging from pure white to red for registered cultivars). This unique floral morphology makes this species highly valued in horticulture, landscaping, and floristry, while its phytochemical profile also indicates potential for pharmaceutical purposes [2]. Despite these qualities, biotechnological research on *L. spectabilis* has been limited, and only a few tissue culture protocols or propagation systems and cryopreservation approaches have been described to date [3].

Cultivation of *L. spectabilis* outdoors is challenging since plants are susceptible to various biotic stressors. Insect pests, slugs, and fungal pathogens (such as *Fusarium* and *Botrytis cinerea* Pers.) can severely impact their growth and survival [4]. Viral infections, which may remain latent (symptomless) in host tissues, also pose a threat [5]. Several viruses are known to infect bleeding heart [6,7,8], and a novel potyvirus (“Lamprocapnos virus A”) was recently identified [9]. Additionally, phytoplasmas (obligate intracellular pathogens transmitted by phloem-feeding insects) can cause flower deformities, yellowing, and overall decline in ornamental value in infected plants [10,11]. These stressors result in serious economic losses. Moreover, the maintenance of in vivo cultivar collections requires intensive agronomic efforts and is becoming increasingly challenging under shifting climate conditions, including more frequent droughts and flooding [12,13,14,15]. Collectively, these obstacles highlight the need to develop reliable, long-term strategies for conserving the genetic resources of *L. spectabilis* cultivars.

Cryopreservation, i.e., the storage of tissues in liquid nitrogen (LN, −196 °C), is currently regarded as the most secure strategy for long-term conservation of plant genetic resources [16]. At this temperature, cell metabolism is suppressed, theoretically allowing indefinite storage without genetic or physiological disruptions, but the efficiency of this method mainly depends on the procedure used for adapting plant tissues to cryogenic storage. One of them is the encapsulation-dehydration method, which preserves meristems by enclosing them in alginate beads and partially dehydrating them before plunging them in LN to prevent ice crystal formation [17,18,19]. For *L. spectabilis*, this protocol was reported to yield survival rates below 40% [20]. Subsequent experiments showed that shoot tip survival is highly dependent on the bead matrix composition, type, and time of plant vitrification solution (PVS) applied. By testing different factors, the method was optimized, and shoot tip survival increased to around 68% [21]. Nevertheless, cryobiology studies still require searching for novel protective agents to simplify the procedure and/or increase their effectiveness.

While the efficiency of cryopreservation protocols is often measured by survival and regrowth, the long-term value of conserved material also depends on genetic and physiological stability. Plants after cryopreservation may exhibit somaclonal variation, DNA damage, or altered photosynthetic performance, which affect their development [22,23,24]. For example, in LN-recovered strawberry plants (*Fragaria* × *ananassa* Duch.), there were no significant differences in most of the characteristics studied, but a few traits, such as sugar content and pH of fruits, showed higher values after cryostorage compared to glasshouse-grown plants [25]. Among physiological traits, the efficiency of photosystem II (PSII) is considered a sensitive indicator of plant quality and health [26]. Chlorophyll *a* fluorescence (ChF) signal analysis is a popular, rapid, non-invasive technique for studying the photosynthetic process on a foliar scale under stress regimes [27]. Hazrati et al. observed that water and light stress resulted in an increased initial fluorescence (F_0_) and decreased maximum fluorescence (F_m_) in *Aloe vera* L [28]. Changes in chlorophyll *a* fluorescence parameters during cold stress, resulting in overall downregulation of PSII-related specific energy fluxes, were also reported in maize (*Zea mays* L.) and soybean (*Glycine max* [L.] Merrill) [29]. Therefore, assessments of PSII function provide valuable insights into the quality of post-cryopreservation plant material and should be monitored [30,31].

Recent studies have explored the use of exogenous protectants, such as nanoparticles (NPs) and biostimulants, to enhance plant recovery following cryopreservation. Nanoparticles, including zinc oxide (ZnO) and silver (Ag) nanoparticles, have been shown to mitigate oxidative stress, boost antioxidant defenses, and improve membrane stability [32]. Gold nanoparticles (Au NPs) have also been demonstrated to improve plant growth and stress resistance [33]. Additionally, melatonin (MEL; N-acetyl-5-methoxytryptamine), a well-known antioxidant and signaling molecule, has demonstrated protective effects on plant tissues under various (a)biotic stresses, including during cryopreservation [34,35,36]. It acts as a stress-relieving hormone and as a bio-stimulator of plant growth by improving gene expression, enzyme activity, and controlling redox equilibrium [37]. Uchendu et al. demonstrated that melatonin added to the preculture and recovery media enhanced the recovery of cryopreserved shoot tips of American elm (*Ulmus americana* L.) by approximately 20–30% [38]. Likewise, the survival rate of cryopreserved callus of *Rhodiola crenulata* (Hook.f. & Thomson) H. Ohba increased after pretreatment for 5 days with 0.1 μM melatonin prior to freezing in liquid nitrogen [39]. Nanoparticles and melatonin were successfully used in regulating oxidative stresses, lipid peroxidation, and DNA fragmentation to protect animal gametes and embryos during vitrification [40]. Iron oxide (Fe_3_O_4_) magnetic NPs improved the vitrification of mouse immature oocytes and modulated the pluripotent genes in derived pronuclear-stage embryos [41]. However, to date, there is no information on the fortification of traditional plant vitrification solutions with NPs or MEL.

Therefore, the aim of this preliminary study was to analyze the genetic stability and photosystem II functioning of *L. spectabilis* plants regenerated from cryopreserved synthetic seeds after vitrification in the presence of NPs or melatonin in PVS. The hypothesis assumed that the addition of specific nanoparticles or melatonin at optimized concentrations during cryopreservation can enhance the cryopreservation efficiency in terms of plant recovery and maintaining the genetic stability of recovered microshoots of *L. spectabilis*.

## 2. Results

### 2.1. Effect of Nanoparticles and Melatonin on the Explant Recovery

**Figure 1:**
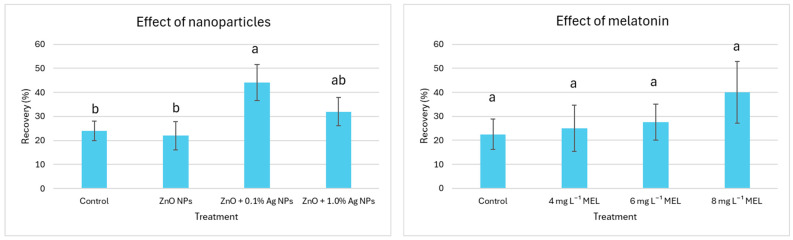
Effect of nanoparticles (NPs) and melatonin (MEL) added into the plant vitrification solution (PVS3) during the encapsulation-vitrification cryopreservation protocol on the explant recovery in *L. spectabilis* ‘Valentine’. The same lowercase letters above the mean ± error bars indicate no significant (*p* ≤ 0.05) differences according to a one-way ANOVA test and Duncan’s multiple range test.

The supplementation of PVS3 with ZnO + 0.1% Ag NPs resulted in a significant increase in explant recovery potential (44%) compared to the control (24%) and ZnO + 1.0% Ag NPs (22%). The application of ZnO + 1.0% Ag NPs also yielded good results (32% recovery), although it was not significantly different from the other treatments and control (Figure 1).

There was no significant effect of melatonin on the explant recovery post LN-storage, especially if applied at lower concentrations (4 and 6 mg L^−1^; recovery: 25–27.5%); however, there was a tendency suggesting that a higher (8 mg L^−1^) level of MEL increased the recovery potential (40%) (Figure 1).

### 2.2. Effect of Nanoparticles and Melatonin on Chlorophyll a Fluorescence

There was a significant effect of the studied factors on the chlorophyll *a* fluorescence in *L. spectabilis* ‘Valentine’ (Table 1). In the experiment with nanoparticles, the application of ZnO NPs in a pure form and combined with 0.1% Ag NPs resulted in the highest F_0_ values. In contrast, the lowest value was recorded with ZnO + 1.0% Ag NPs. On the other hand, supplementation of PVS3 with ZnO + Ag NPs resulted in higher F_v_ and F_m_ parameters compared to other treatments. The values of F_v_/F_m_ and F_v_/F_0_ ratios were significantly enhanced by ZnO NPs + 1.0% Ag NPs (0.67 and 2.16, respectively), followed by ZnO + 0.1% Ag NPs (0.52/1.12), whereas the control had the lowest values (0.38 and 0.70).

As for the second experiment, the addition of 4 mg L^−1^ MEL into PVS3 resulted in the highest initial chlorophyll fluorescence. On the other hand, the use of 6 mg L^−1^ MEL significantly increased the following parameters: F_v_, F_m_, as well as F_v_/F_m_ (0.61) and F_v_/F_0_ (1.71) compared to the control (0.48; 1.07) (Table 1).

### 2.3. Effect of Nanoparticles and Melatonin on the Genetic Integrity of Plants

**Figure 2:**
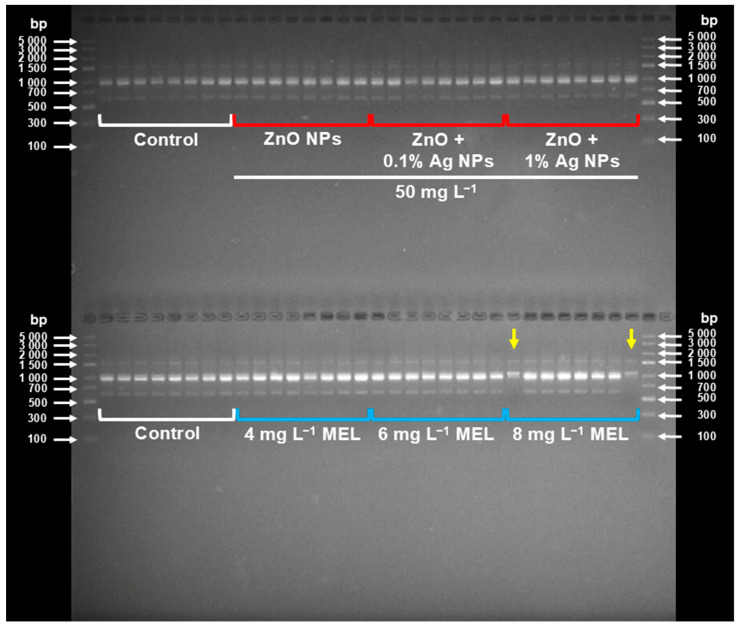
Gel electrophoresis of PCR-SCoT amplified products using S6 primer in *L. spectabilis* ‘Valentine’ plants treated with zinc oxide (ZnO) and silver (Ag) nanoparticles (NPs; upper line) and melatonin (MEL; bottom line) during the vitrification step of the cryopreservation procedure. The outermost lanes serve as DNA base pair (bp) weight markers. The polymorphic band patterns are indicated with yellow arrows.

Among the eight primers used, primer S8 generated the highest number of amplicons (9 per sample), while primer S2—the lowest (1 per sample) (Table 2). Only one primer (S6) detected two polymorphic plants, i.e., 3% of all plants studied. These specimens had the same genotype and were both from the treatment PVS3 + 8 mg L^−1^ MEL (Figure 2).

The analysis of molecular variance (AMOVA) revealed that 3% of the total variation could be attributed to differences between populations (melatonin-caused), while 97% of the variation was of intraspecific origin. Nevertheless, the PhiPT value was 0.034, but it was not statistically significant (*p* = 0.345), suggesting a lack of clear genetic differentiation between the analyzed populations.

## 3. Discussion

In our previous research, we studied the effects of Ag NPs, Au NPs, and ZnO NPs (at concentrations of 5–15 ppm) added either to the preculture medium or the alginate matrix during the encapsulation–vitrification protocol. We concluded that the cryopreservation efficiency in bleeding heart depends not only on the type and timing of application but also on the nanoparticle concentration [31]. In the present study, we aimed to verify the effect of supplementing the PVS solution with novel nanomaterials (ZnO–Ag nanocomposites). Since the exposure time to NPs was much shorter (150 min) compared to our previous experiments (where preculture lasted 7 days and NPs in the alginate matrix were available to explants throughout the recovery step), we decided to increase their concentration to 50 mg L^−1^, to increase the chance of nanoparticles penetrating the alginate bead. The choice of melatonin concentration was based on the available literature [42]. However, previous cryopreservation studies have focused on long-term explant treatments with MEL (0.1–4 µM) during preculture and recovery [38,43]. Therefore, in this study, we used a higher concentration.

### 3.1. Effect of Nanoparticles and Melatonin on Explant Recovery Post LN Storage

Our findings demonstrate that the addition of nanoparticles to the vitrification solution can enhance explant recovery after storage in liquid nitrogen, although the final effect depends on their formulation. Explant recovery was significantly improved with ZnO + 0.1% Ag NP treatment (44%) compared to the control (24%) and pure ZnO NPs (22%) (Figure 1). This suggests that low concentrations of Ag in the nanoparticle mixture may support stress mitigation without having phytotoxic effects. The beneficial effects of nanoparticles in cryopreservation have been previously explained by their capacity to stimulate the antioxidant defence system, which can reduce oxidative stress and cellular damage during vitrification and rewarming processes [44]. Alfosea-Simón et al. [45] described the stress-alleviating properties of Ag NPs, regarding both biotic and abiotic factors. They concluded that these nanomaterials increase the production of bioactive compounds and antioxidants in plants, although the final effect depends on several factors such as the species, type of application, or the properties of the nanoparticle used. Kulus et al. [31] also reported that a lower concentration of Ag NPs in the encapsulation matrix enhanced the cryopreservation efficiency of bleeding heart ‘Valentine’ shoot tips, whereas no positive effect of ZnO NPs in this cultivar was observed. It is possible that the tiny silver particles were able to transport the cryoprotectors inside the cells more effectively than larger ZnO NPs [46]. However, the reduced recovery observed in this study with higher Ag concentration (ZnO + 1.0% Ag NPs) suggests toxicity at elevated levels. This corresponds with the results by Tymoszuk and Kulus [47], who found that the regeneration efficiency of chrysanthemum shoots in vitro declined with the increasing level of Ag NPs in the culture medium. Ag NPs are sometimes considered quite aggressive in plant tissue culture; therefore, their use should be performed with caution, not to exceed the threshold level.

The effect of melatonin on explant recovery was less evident, although it can be suggested that higher concentrations of this compound may have a beneficial effect on the protocol efficiency (Figure 1). The multifunctional character of melatonin, which is involved in the metabolism of indole-3-acetic acid (IAA), cytokinin, ethylene, gibberellin, and auxin carrier proteins [48], can explain the observed beneficial effects here (increase in explant recovery by 18%). Our results corroborate the findings of Uchendu et al., who reported that either vitrification or encapsulation-vitrification methods for the long-term storage of germplasm of medicinal plant species can be improved by adding antioxidant melatonin to both preculture and regrowth media [43]. However, according to Uchendu et al. [38], the beneficial role of MEL was observed only at lower concentrations (0.1–0.5 µM), with little effect at higher doses. These authors, however, used a much longer treatment (even a few weeks in the recovery medium) compared to ours (150 min).

In the present study, lowered concentrations of alginate (2%) were used, compared with the previous studies on bleeding heart cryopreservation [21], for the ease of penetration of the modified vitrification solution and better delivery of NPs and MEL. However, it seems that this resulted in a less effective protection of the explants, reflected by the relatively limited recovery of the plants (below 30% in most cases). Nevertheless, a ≥40% protocol efficacy in the optimal treatments meets the criteria set by most of the world’s gene banks [24].

### 3.2. Effect of Nanoparticles and Melatonin on Plants’ Physiological Condition

The application of pure ZnO NPs and in combination with 0.1% Ag NPs resulted in the highest F_0_ values, which depict the initial fluorescence (i.e., before any light is absorbed by the plant) (Table 1). This increase in F_0_ suggests an altered photosystem II (PSII) dynamics, possibly due to stress-induced changes in the thylakoid membrane structure or the interaction between the nanoparticles and the chloroplasts, all leading to changes in electron transport processes [49,50]. On the other hand, lower values of F_0_ after the application of ZnO + 1.0% Ag NPs compared to the control, suggest a better condition of the PSII. Conversely, supplementation of PVS3 with combined ZnO and Ag NPs resulted in enhanced F_v_ and F_m_ values compared to other treatments. This suggests that while NPs may negatively affect initial fluorescence, they could still have positive effects on the overall functioning of PSII. Our results can be related to the antioxidant properties of nanoparticles, as reported also by Silva et al. [51], which can mitigate oxidative stress during cryopreservation. Importantly, most of the NPs treatments resulted in an increased F_v_/F_m_ and F_v_/F_0_ ratios, indicating better energy transfer through PSII and improved photochemistry. Even though ZnO NPs alone did not increase photosynthesis, they also did not cause negative effects.

As for melatonin, the addition of 4 mg L^−1^ MEL in PVS3 resulted in the highest F_0_ values, which could result in an initial stimulation of chloroplasts, possibly due to the antioxidant properties of melatonin [52] (Table 1). More importantly, the application of 6 mg L^−1^ MEL resulted in a significant increase in F_v_, F_m_, as well as the ratios F_v_/F_m_ and F_v_/F_m_ compared to the control. This highlights that melatonin is an effective agent in plant tissue culture even at low concentrations. In accordance with our research, Kuppusamy et al. [53] reported that the exogenous application of MEL enhanced the photosynthesis of Mung bean under drought and high-temperature stress conditions.

Nevertheless, it should be pointed out that the optimal F_v_/F_m_ range in most plant species is approximately between 0.79 and 0.84 [54]. Therefore, the obtained lower values (0.38–0.67) indicate moderate (≥0.60) or even profound stress (<0.60) and potential damage in plants that could explain the low recovery rates in most treatments (Table 1).

### 3.3. Effect of Nanoparticles and Melatonin on Plants’ Genetic Stability

In the present study, none of the used nanoparticle formulations caused any genetic alterations in the LN-derived plants, as confirmed by eight SCoT primers (Table 2; Figure 2). This is most important as maintaining stability is crucial when developing any (long-term) storage protocol. Nanoparticles are known to have the capability to penetrate the plant cell and interact directly with its DNA and accumulate in the nucleus [55,56]. Nowadays, due to their exceptionally small size, nanomaterials are even being successfully used for gene transfer to plants [57,58]. Several studies report the genotoxic effect of silver and zinc oxide NPs in plants [59,60]. Tymoszuk and Kulus [47] found that in chrysanthemum, treatment with silver nanoparticles resulted in the occurrence of heritable mutations that manifested as a change in inflorescence shape and color. This mutagenic effect was particularly evident when using high (100 ppm) concentrations. Therefore, we decided to use much lower doses of NPs. Moreover, in the present experiment, nanoparticles were only added to the PVS solution, and the explants were embedded in a protective alginate capsule. Therefore, the tissues did not have direct contact with the NPs, unlike the previous experiments, where nanomaterials were poured directly onto the culture medium or explant surface [47,61]. Moreover, in the case of short-term contact during vitrification (150 min), their effect is probably more protective than mutagenic—especially in the presence of a protective coating.

Studies using the Ames assay and the single cell gel electrophoresis (SCGE or COMET) assay on mammalian cells have shown that melatonin itself is not mutagenic. No mutations were induced in the Ames assay, and only low clastogenic activity was demonstrated in the COMET assay at very high concentrations (100 µM MEL). On the contrary, it was even found that it protected the cells against genetic damage induced by various mutagens [62]. As for plants, melatonin affected DNA methylation and gene expression in grape berries, although no classical mutagenic effects (changes in DNA sequence) have been reported [63]. Indeed, two specimens were found polymorphic in our SCoT analysis, but they had an identical banding pattern, and the statistical analysis excluded the role of melatonin in this process (Table 2). According to Kulus et al. [64], bleeding heart undergoes somaclonal variation quite easily. It is most probable that the polymorphisms discovered here were related to excessive cryoinjury (reflected by the low F_v_/F_m_ values) [64].

## 4. Materials and Methods

### 4.1. Preculture of Explants

**Figure 3:**
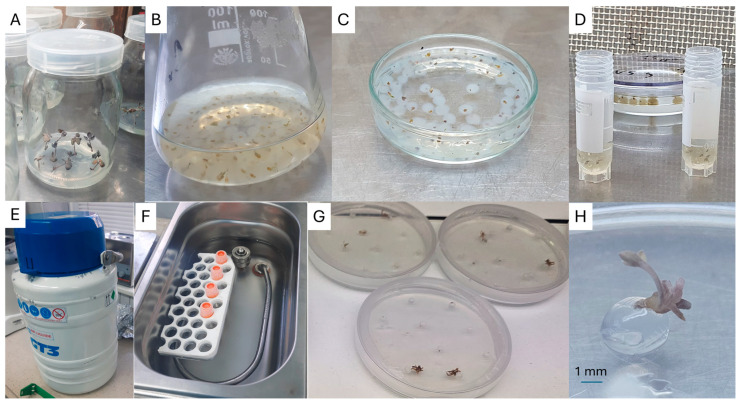
Cryopreservation procedure of *L. spectabilis* ‘Valentine’: (**A**)—preculture, (**B**)—bead polymerization, (**C**)—vitrification in PVS, (**D**)—beads in cryovial, (**E**)—storage in Dewar flask, (**F**)—rewarming, (**G**,**H**)—shoots recovering in vitro after five weeks of culture.

In vitro-derived single-node explants of *L. spectabilis* ‘Valentine’ were cultured for one week on the solid MS medium [65] with 9% (*w*/*v*) sucrose, 4.65 μM kinetin, and 10 μM of abscisic acid according to Kulus et al. [66] (Figure 3A). At least 40 explants per treatment were used. The cultures were kept in a growth room at 23 °C ± 1 °C, under 16 h photoperiod conditions and photosynthetic photon flux density of approximately 30 μmol m^−2^ s^−1^ provided by standard cool daylight TLD 54/36W fluorescent tubes (Koninklijke Philips Electronics N.V., Eindhoven, The Netherlands). The obtained shoot tips were used in the cryopreservation experiments.

### 4.2. Cryopreservation Experiment

#### 4.2.1. Effect of NPs and Melatonin Added into the PVS

Plant vitrification solution 3 (PVS3) was prepared as a 50% (*w*/*v*) glycerol and 50% (*w*/*v*) sucrose mixture and sterilized by autoclaving. Two independent sets of experiments were conducted to evaluate the effect of PVS3 modification on cryopreservation efficiency: (i) supplementation with nanoparticles and (ii) supplementation with melatonin.

#### Nanoparticle-Supplemented PVS3

Three nanoparticle formulations were tested at the same final concentration of 50 mg L^−1^: (1) ZnO NPs (1.5% H_2_O), (2) ZnO + 0.1% Ag NPs (1.5% H_2_O), and (3) ZnO + 1% Ag NPs (1.5% H_2_O). The NP mixtures were synthesized using the microwave–solvothermal co-synthesis method [67,68] and characterized in detail by Tymoszuk et al. [69,70] and Pokrowiecki et al. [71]. For proper dispersion, each NPs–PVS3 solution was subjected to ultrasonication for 30 min in an Elmasonic S80(H) Ultrasonic Cleaner (37 kHz, 150 W; Elma Schmidbauer GmbH, Singen, Germany). Pure PVS3 served as a reference group.

#### Melatonin-Supplemented PVS3

In a separate experiment, melatonin (Sigma-Aldrich, Darmstadt, Germany) was added to the PVS3 solution at concentrations of 4, 6, and 8 mg L^−1^ (corresponding to 17, 25.5, and 34 µM, respectively). The melatonin-modified PVS3 served as an alternative treatment to NP-enriched solutions. As the reference group, pure PVS3 was used.

### 4.3. Encapsulation of Explants, Dehydration, and LN Storage

Excised shoot tips were embedded for 10 min in 2% (*w*/*v*) sodium-alginate (Carlo Erba, Val-de-Reuil, France) based on MS medium salts, without calcium chloride (CaCl_2_), supplemented with 9% sucrose. Then, the beads were hardened in 0.1 M CaCl_2_ solution for 30 min (Figure 3B). The encapsulated explants were rinsed thrice with distilled sterile water. The beads were osmo-protected with the loading solution (2.0 M glycerol and 0.4 M sucrose) for 20 min. Next, the beads were dehydrated for 150 min with PVS3, modified as described above, placed in a 2.0 mL sterile cryovial, and immersed in LN for 15 min (Figure 3C–E).

### 4.4. Rewarming and Plant Recovery

After LN storage, cryovials were rapidly rewarmed in a water bath (39 ± 1 °C) for 3 min (Figure 3F). The explants were rinsed with washing solution (WS; liquid MS medium with 1.2 M sucrose) for 30 min. WS was replaced with a fresh portion after half-time exposure (15 min), and then synthetic seeds were inoculated on a Petri dish with MS recovery medium with 3% sucrose and 8.0 g L^−1^ agar. Petri dishes, sealed with parafilm, were kept for 48 h at 23 ± 1 °C in darkness and then transferred to the same conditions as the in vitro stock plants (Figure 3G,H). After 6 weeks, the share of viable explants (i.e., showing signs of growth) was assessed, and they were transferred to a fresh PGR-free MS medium for further growth under conditions (of growth room) same as described above for preculture of explants (Section 4.1).

### 4.5. Chlorophyll a Fluorescence

The level of stress was measured based on the maximum efficiency of photosystem II (PSII) in leaves of at least 10 in vitro-grown plants per experimental variant. Before the measurement, the leaves were adapted to darkness for 30 min. The fluorescence kinetics of chlorophyll *a* were measured using a portable plant stress meter OS30p+ (Opti-Sciences Inc., Hudson, NH, USA), and then initial fluorescence (F_0_), variable fluorescence (F_v_), and maximum fluorescence (F_m_) were determined. The F_v_/F_m_ (1) ratio indicates the maximum quantum yield of PSII, while F_v_/F_0_ (2) reflects its potential activity. Their ratios (F_v_/F_m_ and F_v_/F_0_) were calculated using formulas provided by Roháček [72] and expressed in relative units:

F_v_/F_m_ = (F_m_ − F_0_)/F_m_ (1)

F_v_/F_0_ = (F_m_ − F_0_)/F_0_ (2)

Lower F_0_ and higher F_m_, F_v_, F_v_/F_m_, and F_v_/F_0_ values indicate better photosynthetic function, since stress usually causes F_0_ to rise and the other parameters to decline [73].

### 4.6. Genetic Stability Evaluation

The genetic fidelity of in vitro-grown plants was assessed using SCoTs as described by Collard and Mackill [74]. A total of 64 individuals (8 from each experimental variant) were used in this study.

Total genomic DNA was isolated from fresh tissues using a Genomic Mini AX Plant Spin kit (A&A Biotechnology, Gdynia, Poland), according to the manufacturer’s instruction. Matrices with high-quality parameters, measured using a NanoPhotometer NP80 (Implen, Munchen, Germany), were used in further studies. The isolated DNA was stored at 4 °C.

A total of 8 primers (Genomed, Warsaw, Poland) were used for the polymerase chain reaction (PCR) (Table 3). PCR was performed in a 25-µL reaction solution containing 2×PCR Master Mix Plus kit (A&A Biotechnology, Gdynia, Poland). DNA amplification was performed in a BioRad C1000 Touch thermal cycler (Bio-Rad, Hercules, CA, USA) using the profile described in Kulus et al. [56]. The amplified DNA fragments were separated on a 1.5% agarose gel (Blirt, Gdańsk, Poland) in a TBE (Tris—borate—EDTA) buffer (Biometra P25, Jena, Germany) and detected by staining with SimplySafe™ (EURx, Gdańsk, Poland). Gel images were recorded using a GelDoc XR+ Gel Photodocumentation System UV transilluminator with the Image Lab 4.1 software (Bio-Rad, Hercules, CA, USA). Molecular weights of the fragments were estimated using a 100–5000 bp DNA molecular marker (GPB5000bp DNA Ladder, GenoPlast Biochemicals, Rokocin, Poland).

The banding patterns were recorded as a 0–1 binary matrix, where “1” indicates the presence and “0” the absence of a given fragment, followed by statistical analysis.

### 4.7. Statistical Analysis

The obtained results were statistically analyzed through one-way analysis of variance (ANOVA), and means were compared with Duncan’s multiple range test (*p*  ≤  0.05), using Statistica 13.3 software (StatSoft, Tulsa, OK, USA). For data expressed as a percentage, the arcsin transformation was used. The significance of the detected genetic variation was verified through analysis of molecular variance (AMOVA) using GeneAlex 6.5 software [75]. For this purpose, the PhiPT (ΦPT) value was calculated to quantify genetic differentiation among populations based on molecular data (3), with the assumption that control, NPs-, and MEL-treated plants represent three different populations.

(3) $$\mathrm{PhiPT}=\frac{\mathrm{Variance}\;\mathrm{among}\;\mathrm{populations}}{\mathrm{Total}\;\mathrm{variance}}$$

PhiPT values range from 0 to 1, where 0 indicates no genetic differentiation (populations are genetically similar) and 1 indicates complete genetic differentiation (populations are entirely distinct).

## 5. Conclusions

The present study confirmed the validity of PVS supplementation with NPs and melatonin. Interestingly, the treatment that resulted in the highest explant recovery (PVS3 with ZnO + 0.1% Ag NPs and PVS3 with 8 mg L^−1^ MEL) was not reflected by the highest photosynthetic activity of recovered plants, suggesting that explant survival and PSII functionality are regulated by different physiological mechanisms. Protective substances can temporarily slow down metabolism, which allows the plant to survive stress (e.g., by limiting ROS production). It is possible that after cryostorage, the plants undergo a lag phase and need more time for plastid regeneration. Importantly, the applied treatments did not induce significant genetic alterations, which is crucial in cryopreservation procedures. Therefore, optimized protocols could be applied in gene banks for the storage of germplasm of *L. spectabilis*, e.g., for breeding purposes. Further research should focus on the simultaneous application of NPs and MEL or using other nanomaterials, such as apatite or hydroxyapatite, as carriers of melatonin.

## Figures and Tables

**Table 1:** Effect of nanoparticles (NPs) and melatonin (MEL) added into the plant vitrification solution (PVS3) during the encapsulation-vitrification cryopreservation protocol on the chlorophyll *a* fluorescence parameters in the leaves of *L. spectabilis* ‘Valentine’. The same lowercase letters (for each fluorescence parameter, separately) indicate no significant (*p* ≤ 0.05) differences according to the one-way ANOVA test and Duncan’s multiple range test.

Treatment	Fluorescence Parameters				
	F_0_	F_v_	F_m_	F_v_/F_m_	F_v_/F_0_
	Nanoparticles in PVS3				
Control	170.05 ± 11.59 b	114.35 ± 19.35 b	284.40 ± 26.08 c	0.38 ± 0.03 c	0.70 ± 0.09 c
ZnO NPs	203.70 ± 9.64 a	143.05 ± 11.43 b	346.75 ± 19.69 bc	0.41 ± 0.01 c	0.70 ± 0.04 c
ZnO + 0.1% Ag NPs	212.45 ± 13.94 a	246.80 ± 23.87 a	459.25 ± 36.07 a	0.52 ± 0.01 b	1.12 ± 0.07 b
ZnO + 1.0% Ag NPs	137.40 ± 7.53 c	280.20 ± 14.01 a	417.60 ± 16.37 ab	0.67 ± 0.02 a	2.16 ± 0.18 a
	Melatonin in PVS3				
Control	152.10 ± 7.82 b	162.65 ± 22.51 b	314.75 ± 22.51 b	0.48 ± 0.03 b	1.07 ± 0.15 b
4 mg L^−1^ MEL	188.55 ± 16.56 a	200.20 ± 21.21 ab	388.75 ± 21.21 ab	0.50 ± 0.03 b	1.18 ± 0.14 b
6 mg L^−1^ MEL	148.10 ± 8.34 b	249.25 ± 19.81 a	397.35 ± 19.81 a	0.61 ± 0.02 a	1.71 ± 0.14 a
8 mg L^−1^ MEL	153.20 ± 10.66 b	194.00 ± 22.21 ab	347.20 ± 22.21 ab	0.53 ± 0.04 ab	1.37 ± 0.16 ab

**Table 2:** PCR products obtained from *L. spectabilis* ‘Valentine’ with a start codon targeted polymorphism (SCoT) marker system.

No.	Primer Sequence 5′➞3′	No. of Bands	No. of *Loci*				Share of Polymorphic *Loci* [%]	No. (%) of Polymorphic Plants	No. of Genotypes
			∑	Mono.	Poly.	Spec.			
S1	CAA CAA TGG CTA CCA CCG	512	8	8	0	0	0	0	1
S2	CAA CAA TGG CTA CCA CCT	64	1	1	0	0	0	0	1
S3	CAA CAA TGG CTA CCA CGT	320	5	5	0	0	0	0	1
S4	ACG ACA TGG CGA CCA ACG	192	3	3	0	0	0	0	1
S5	ACG ACA TGG CGA CCA TCG	448	7	7	0	0	0	0	1
S6	ACC ATG GCT ACC ACC GTG	254	5	2	3	0	60	2 (3%)	2
S7	CCA TGG CTA CCA CCG CCA	128	2	2	0	0	0	0	1
S8	CCA TGG CTA CCA CCG CAG	576	9	9	0	0	0	0	1

mono.—monomorphic (present in all specimens), poly.—polymorphic (present in more than one specimen but not in all), spec.—specific (present in a single specimen).

**Table 3:** List of markers used in the SCoT analysis designed according to Collard and Mackill [74].

No.	Marker	Sequence (5′-3′)	%GC
S1	SCoT3	CAACAATGGCTACCACCG	56
S2	SCoT4	CAACAATGGCTACCACCT	50
S3	SCoT8	CAACAATGGCTACCACGT	50
S4	SCoT12	ACGACATGGCGACCAACG	61
S5	SCoT13	ACGACATGGCGACCATCG	61
S6	SCoT25	ACCATGGCTACCACCGGG	67
S7	SCoT26	ACCATGGCTACCACCGTC	61
S8	SCoT28	CCATGGCTACCACCGCCA	67

## Data Availability

The data presented in this study are available from the corresponding author upon reasonable request.

## Abbreviations

The following abbreviations are used in this manuscript:

ChF	Chlorophyll fluorescence
IAA	Indole-3-acetic acid
LN	Liquid nitrogen
MEL	Melatonin
MS	Murashige and Skoog
NPs	Nanoparticles
PCR	Polymerase chain reaction
PSII	Photosystem II
PGR	Plant growth regulator
PVS	Plant vitrification solution
ROS	Reactive oxygen species
SCoT	Start Codon Targeted Polymorphism
WS	Washing solution
ΦPT	Proportion of genetic variation among populations relative to total variation
